# Identification of Comorbidities in Obstructive Sleep Apnea Using Diverse Data and a One-Dimensional Convolutional Neural Network

**DOI:** 10.3390/s26031056

**Published:** 2026-02-06

**Authors:** Kristina Zovko, Ljiljana Šerić, Toni Perković, Ivana Pavlinac Dodig, Renata Pecotić, Zoran Đogaš, Petar Šolić

**Affiliations:** 1 Faculty of Electrical Engineering, Mechanical Engineering and Naval Architecture, University of Split, 21000 Split, Croatia; ljiljana@fesb.hr (L.Š.);; 2 Department of Neuroscience, School of Medicine, Sleep Medicine Center, University of Split, 21000 Split, Croatia; ivana.pavlinac@mefst.hr (I.P.D.);

**Keywords:** obstructive sleep apnea (OSA), deep learning (DL), 1D-CNN, multi label classification (MLC), multi label confusion matrix (MLCM), sleep medicine, polysomnography

## Abstract

Recent advances in deep learning (DL) have enabled the integration of diverse biomedical data for disease prediction and risk stratification. Building on this progress, the overall objective of this study was to develop and evaluate a multimodal DL framework for robust multi-label classification (MLC) of major comorbidities in patients with obstructive sleep apnea (OSA) using physiological time series signals and clinical data. This study proposes a robust framework for multi-label classification (MLC) of comorbidities in patients with OSA using diverse physiological and clinical data sources. We conducted a retrospective observational study including a convenience sample of 144 patients referred for overnight polysomnography at the Sleep Medicine Center (SleepLab Split), University Hospital Centre Split (KBC Split), Split, Croatia. Patients were selected based on predefined inclusion criteria and data availability. A one-dimensional Convolutional Neural Network (1D-CNN) was developed to process and fuse time series signals, oxygen saturation ($SpO_{2}$), derived $SpO_{2}$ features, and nasal airflow (FP0), with demographic and physiological parameters, enabling the identification of key comorbidities such as arterial hypertension, diabetes mellitus, and asthma/COPD. The instruments included polysomnography-derived signals (SpO_2_ and FP0 airflow) and structured demographic/physiological parameters. Signals were preprocessed and used as inputs to the proposed fusion model. The proposed model was trained and fine-tuned using the Optuna hyperparameter optimization framework, addressing class imbalance through weighted loss adjustments. Its performance was comprehensively assessed using multi-label evaluation metrics, including macro/micro F1-score, AUC-ROC, AUC-PR, subset and partial accuracy, Hamming loss, and multi-label confusion matrix (MLCM). The study protocol was approved by the Ethics Committee of the School of Medicine, University of Split (Approval No. 003-08/23-03/0015, Date: 17 October 2023). The 1D-CNN achieved superior predictive performance compared to traditional machine learning (ML) classifiers with macro AUC-ROC = 0.731 and AUC-PR = 0.750. The model demonstrated consistent behavior across age, gender, and BMI groups, indicating strong generalization and minimal demographic bias. In conclusion, the results confirm that $SpO_{2}$ and airflow signals inherently encode comorbidity-specific physiological patterns, enabling efficient and scalable screening of OSA-related comorbidities without the need for full polysomnography. Although the study is limited by data set size, it provides a methodological basis for the application of multi-label DL models in clinical decision support systems. Future research should focus on the expansion of multi-center datasets, thereby improving model interpretability and potential clinical adoption.

## 1. Introduction

Obstructive sleep apnea (OSA) is one of the most common sleep-related breathing disorders and affects a large portion of the global population. Current estimates suggest that nearly one billion individuals have some degree of OSA, which highlights its significance as a major public health concern [1,2]. OSA is characterized by repeated pauses or reductions in airflow during sleep due to upper airway obstruction. These events cause oxygen desaturation, fragmented sleep, and increased physiological stress. When these disturbances occur chronically, they can lead to serious long-term cardiovascular, metabolic, and respiratory consequences [3,4].

Thus, extensive research has shown that OSA is strongly associated with several important comorbidities, including hypertension [5], type 2 diabetes mellitus [6], and chronic respiratory diseases such as asthma or chronic obstructive pulmonary disease (COPD) [7,8]. The likelihood of developing these conditions increases with OSA severity. In addition, demographic and clinical factors such as age, body mass index (BMI), metabolic irregularities, and inflammatory processes further influence the interaction between OSA and its comorbidities [9,10]. Detecting these comorbidities early is essential for preventing complications and improving patient outcomes.

Polysomnography (PSG) remains the clinical gold standard for OSA diagnosis. PSG includes continuous monitoring of several physiological signals, such as oxygen saturation ($SpO_{2}$), airflow, heart activity, brain waves, and muscle tone. Although PSG is highly reliable, it is resource intensive, costly, and time consuming. The interpretation of PSG data typically requires manual scoring performed by trained clinicians, which can introduce variability, slow down the diagnostic process, and limit scalability [11,12,13,14]. As the volume and complexity of biomedical data continue to grow, traditional manual analysis becomes increasingly challenging.

Advances in machine learning (ML) and deep learning (DL) have opened new possibilities for improving diagnostic support in sleep medicine. DL models are capable of automatically learning patterns from physiological time series data and often achieve higher performance than traditional analytic methods. Convolutional Neural Networks (CNNs) are particularly effective for analyzing biomedical signals such as $SpO_{2}$ and nasal airflow FP0 because they can extract meaningful temporal features directly from raw input data [14]. These advantages make DL promising for building automated systems aimed at identifying OSA-related health risks.

Most existing research focuses primarily on detecting, monitoring, or classifying the severity of OSA itself, without addressing the broader clinical presentation in which multiple health conditions often occur together [15]. Studies in this field mainly concentrate on identifying apnea and hypopnea events or estimating OSA severity levels, while very few investigate the prediction or classification of comorbidities associated with OSA [16,17]. To the best of current knowledge, only one study highlights the importance of identifying comorbidities in this patient population, and no DL approaches have been developed specifically for multi-label classification (MLC) comorbidity. This gap indicates a clear need for models that can analyze diverse physiological and clinical features in order to detect several coexisting conditions more accurately. The aim of this study is to develop a Deep Neural Network (DNN)-based approach for multi-label classification of OSA-related comorbidities using different types of data, including PSG signals, clinical variables, and signal-derived features.

In this study, a DL-based method using PSG signals and additional clinical information is explored to identify several comorbidities associated with OSA. In the next sections of this paper, the dataset and preprocessing steps applied to the physiological signals will be described. The extraction and preparation of clinical and signal-derived features will be explained. The architecture of the proposed one-dimensional Convolutional Neural Network (1D-CNN) for MLC will be presented, the evaluation metrics and comparison procedures will be outlined, and the experimental results and interpretation of model performance will be discussed. This structure provides a complete overview of how DL can support more accurate and efficient identification of comorbidities in patients with OSA. The general objective of this study is to develop and evaluate a multimodal DL framework for multi-label identification of major comorbidities in patients with obstructive sleep apnea by integrating $SpO_{2}$, nasal airflow (FP0), and structured clinical parameters. The specific objectives are to preprocess physiological signals and derive representative features, design a fusio-based 1D-CNN for multi-label prediction, evaluate performance using established multi-label metrics in comparison with baseline approaches, and assess robustness across demographic subgroups. We hypothesize that multimodal fusion of $SpO_{2}$, FP0, and clinical features improves multi-label comorbidity identification compared with single modality inputs and traditional baseline models, particularly in threshold independent metrics under class imbalance.

## 2. Related Work

Artificial intelligence (AI) techniques are increasingly used in medicine as the availability of large and diverse datasets grows and as clinical practice demands faster and more accessible diagnostic solutions. Different biomedical modalities, including physiological signals, medical imaging, wearable sensor data, and electronic health records, require analytical methods capable of capturing their temporal, spatial, and structural patterns. While traditional ML approaches remain valuable for structured and interpretable data, modern DL architectures such as CNNs, RNNs, LSTMs, and Transformers dominate contemporary research due to their ability to model complex patterns in signals, images, and multimodal inputs [18]. Nevertheless, despite these advances, many existing AI studies remain largely monomodal, and relatively few address the prediction or identification of OSA-related comorbidities. In the domain of physiological time series, common approaches include RNNs, LSTMs, GRUs, 1D-CNNs, and ensemble methods such as RF and XGBoost [19], as well as multimodal architectures combining CNN and LSTM models on EEG, ECG, $SpO_{2}$, and airflow signals [20]. More advanced systems integrate physiological signals and EHR data through hybrid DL/ML frameworks [21], while GAN-based models have been explored for enhancing minority classes in $SpO_{2}$ or airflow datasets [22,23]. Other studies fuse physiological signals with CT imaging or EHR data using CNN–Transformer pipelines [24,25]. Similar methodological diversity is observed in EHR, questionnaire, and population-based clinical research, where Transformers, Graph Neural Networks (GNNs), and hybrid architectures are commonly applied [26,27,28]. In medical imaging, CNNs continue to serve as the foundation, with increasing adoption of Transformers, 3D-CNNs, and hybrid systems that integrate images with physiological or behavioral signals [29,30,31]. Such multimodal solutions often rely on CNN, GNN, and U-Net-based components or combine multiple sensor types through CNN–Transformer frameworks [32,33,34,35]. A separate line of work has investigated multi-label classification with combinations of ECG, EEG, EMG, MRI, CT, and wearable sensor data using SVM, GAN, or reinforcement learning approaches [36,37,38]. AI has also become increasingly prominent in sleep medicine, particularly for automated detection of OSA. Early studies relied on handcrafted features extracted from $SpO_{2}$ or ECG signals processed with Fully Connected Neural Networks (FCNNs) or classical ML methods [39,40]. More recent research employs ResNet models, contrastive learning, multiscale architectures, and attention mechanisms to detect apnea events or estimate AHI directly from physiological signals [41,42,43]. EEG-based and multimodal systems combine wavelet-based features, CNNs, BiLSTMs, and attention models to improve event detection and sleep staging [44,45,46,47,48,49]. Additional OSA-related work integrates anatomical imaging, acoustic data, or thermal and depth information using CNN-based architectures [50,51,52,53]. Clinical variable models using logistic regression (LR), XGBoost, SVM, and RF also remain widely used [54,55,56], together with an increasing emphasis on explainable AI (XAI) for improved clinical trust [57]. Within this broader landscape, prior work specifically targeting OSA comorbidity prediction has mostly focused on predicting individual comorbid conditions or clinically relevant outcomes using ML models trained on demographic, clinical, and sleep-derived descriptors. For instance, ref. [58] addressed OSA-related hypertension prediction by benchmarking multiple ML classifiers, including LR, gradient boosting-based methods (e.g., GBM/XGBoost), ensemble techniques, and a multilayer perceptron, in a cohort of 1493 OSA patients, and further interpreted the best-performing models using permutation importance and SHAP to highlight the relevance of demographic characteristics (e.g., age, BMI) and oxygenation-related measures (e.g., minimum $SpO_{2}$, time below 90%). Their best-performing model, GBM, achieved strong discrimination (AUC-ROC = 0.873) and identified key contributors such as family history of hypertension and the percentage of time with $SaO_{2}<90\%$. Ref. [59] extended comorbidity prediction beyond cardiometabolic risk by developing depression risk models in OSA-Hypopnea Syndrome patients, comparing traditional approaches such as LR and Least Absolute Shrinkage and Selection Operator (LASSO) regularization with tree-based models (Random Forest (RF)), demonstrating that the combination of clinical factors and lifestyle variables can improve stratification of mental health comorbidity. In addition, ref. [60] proposed an ML-enhanced framework for predicting incident atrial fibrillation in patients with concurrent type 2 diabetes and OSA syndrome, integrating ML-based risk modeling with clinical predictors and showing that metabolic indices alongside sleep-disordered breathing severity contribute to cardiovascular comorbidity development. Finally, ref. [61] demonstrated the applicability of ML to long-term prognostic modeling by using an RF predictor with feature selection to estimate 10-year cardiovascular disease-related mortality risk in an OSA cohort, illustrating how data-driven models can capture clinically meaningful outcome risk beyond cross-sectional comorbidity status.

Overall, most existing studies address single comorbidities or long-term outcomes using predominantly tabular predictors, whereas fewer works explore multi-label prediction across multiple comorbidities simultaneously. Moreover, despite rapid progress in AI for sleep medicine, OSA-related research still mainly focuses on estimating AHI, detecting apnea and hypopnea events, or assessing disease severity. As a result, the systematic classification of OSA-related comorbidities remains largely underexplored, and many proposed solutions rely on a single data modality, whether imaging, physiological time series, or clinical metadata, thereby overlooking important cross-modal relationships and interactions. In contrast, the proposed approach formulates comorbidity identification as a multi-label task and integrates signal-derived representations with additional clinical parameters, enabling direct assessment of the contribution of non-signal features through comparison with a signal-only baseline model. This combination addresses a clear methodological gap by moving beyond monomodal, single-outcome modeling toward clinically relevant multi-label comorbidity prediction in OSA.

## 3. Data Description and Preprocessing

### 3.1. Dataset Description

This study was designed as a retrospective observational analysis conducted at the SleepLab, KBC Split, Croatia. The study protocol was approved by the Ethics Committee of the School of Medicine, University of Split (Approval No. 003-08/23-03/0015; Date: 17 October 2023). No a priori sample size calculation was performed, as this study represents an exploratory DL model development and evaluation. Therefore, the final sample size was determined by data availability and predefined inclusion and exclusion criteria. A non-probabilistic purposive (criterion-based) sampling approach was applied by first selecting eligible patients with complete physiological recordings and available clinical information required for multi-label comorbidity annotation. When multiple patients met the same eligibility criteria, a random selection was performed to obtain sufficient representation of each target comorbidity for model training.

The dataset used in this study consists of 144 patients who underwent standard overnight polysomnography (PSG) at the SleepLab Split. All recordings were acquired in clinically supervised conditions using full PSG systems, and the data were exported in European data format (.edf) [62]. For the purpose of this study, two physiological signals were selected due to their strong relevance for respiratory analysis: oxygen saturation ($SpO_{2}$) measured via pulse oximetry and nasal airflow (FP0) recorded using a nasal pressure transducer. Both signals capture essential information about respiratory disturbances during sleep. $SpO_{2}$ reflects blood oxygen fluctuations associated with apnea and hypopnea events, whereas FP0 reflects airflow amplitude and respiratory cycles. Alongside the time series signals, the dataset includes clinical and demographic information, such as age, gender, BMI, heart rate (HR), and Apnea–Hypopnea Index (AHI). A set of signal-derived features was also computed to describe the timing and severity of respiratory events, including the duration of airflow cessation, desaturation duration, the delay between airflow loss and oxygen decline, and slope-based markers reflecting the dynamics of oxygen drops and recovery.

The dataset used in this study consists of 144 patients who underwent standard overnight polysomnography (PSG) at the SleepLab Split. All recordings were acquired in clinically supervised conditions using a full PSG system (ALICE 6 Diagnostic System [63]), and the data were exported in European data format (.edf) [62]. In addition, standardized questionnaires were administered as part of the clinical assessment, including STOP Bang, the Berlin Questionnaire, the Pittsburgh Sleep Quality Index (PSQI), and the Epworth Sleepiness Scale (ESS). All PSG recordings and clinical data were de-identified prior to analysis by removing direct personal identifiers. The dataset was stored on secure institutional systems with access restricted to authorized research personnel, and the analyses were performed in compliance with applicable data protection regulations. Due to patient privacy and ethical restrictions, the raw data are not publicly available. For the purpose of this study, two physiological signals were selected due to their strong relevance for respiratory analysis: oxygen saturation ($SpO_{2}$) measured via pulse oximetry and nasal airflow (FP0) recorded using a nasal pressure transducer. Both signals capture essential information about respiratory disturbances during sleep. $SpO_{2}$ reflects blood oxygen fluctuations associated with apnea and hypopnea events, whereas FP0 reflects airflow amplitude and respiratory cycles. Alongside the time series signals, the dataset includes clinical and demographic information, such as age, gender, BMI, heart rate (HR), and Apnea–Hypopnea Index (AHI). A set of signal-derived features was also computed to describe the timing and severity of respiratory events, including the duration of airflow cessation, desaturation duration, the delay between airflow loss and oxygen decline, and slope-based markers reflecting the dynamics of oxygen drops and recovery.

**Figure 1:**
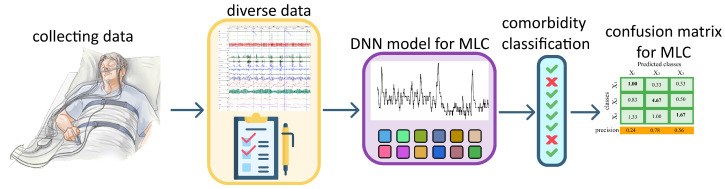
Conceptual workflow of the proposed multi-label comorbidity classification framework in OSA patients. First, overnight PSG data are collected and exported in EDF format. Next, key signals ($SpO_{2}$ and FP0 airflow), derived signal features, and structured clinical parameters are prepared. These multimodal inputs are fused in a multi branch 1D-CNN to simultaneously predict three comorbidities (hypertension, diabetes mellitus, and asthma/COPD). Finally, the model is evaluated using multi-label metrics and the MLCM. (Adapted from [64]).

This multimodal structure provides a comprehensive representation of each patient’s physiological and clinical profile. An illustration of the whole process in the 1D CNN model is illustrated in Figure 1.

### 3.2. Signal Preprocessing

**Figure 2:**
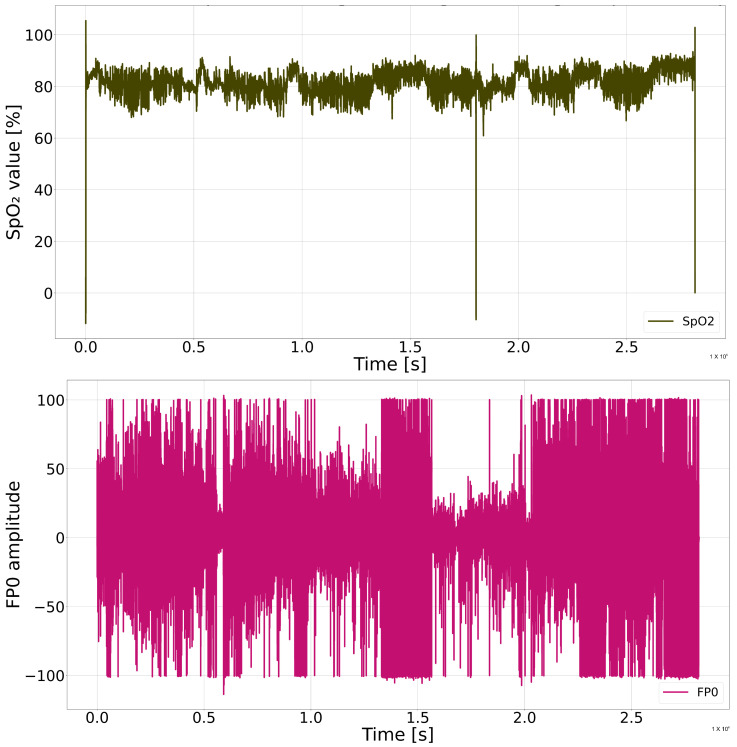
$SpO_{2}$ and FP0 signals of the patient before preprocessing procedure.

The PSG recordings contained noise, artifacts, and varying sampling rates depending on the recording system. Because .edf files occupy a large amount of memory, the $SpO_{2}$ and FP0 channels were extracted from the original PSG recordings and stored in the Feather format to enable faster and more efficient processing [65]. To ensure reliable analysis and allow uniform model input, the signals underwent a structured preprocessing procedure. The original recordings had sampling rates that were much higher than required for analyzing slow respiratory processes. Figure 2 shows $SpO_{2}$ and FP0 signals of one patient during the night before artifact removal and filtering.

Therefore, both $SpO_{2}$ and FP0 signals were resampled to a uniform sampling rate of 5 Hz. This rate is widely used in respiratory signal analysis because it preserves the essential shape of desaturation and airflow events while significantly reducing data volume and computational complexity. The raw signals included invalid values caused by sensor displacement, signal loss, saturation clipping, or patient movement. Such artifacts were detected and corrected using interpolation for short missing segments, replacement of physiologically impossible values, smoothing of extreme spikes, and correction of baseline drift in airflow signals. These steps ensure that only physiologically meaningful patterns remain available for feature extraction and model training. A combination of filtering methods was applied to improve signal smoothness and suppress high-frequency noise: low-pass filtering to preserve slow respiratory components [66], moving average smoothing to stabilize short-term fluctuations [67], and Savitzky–Golay filtering before computing derivatives. These filters help reveal true desaturation patterns and airflow changes while avoiding distortion of clinically relevant events.

**Figure 3:**
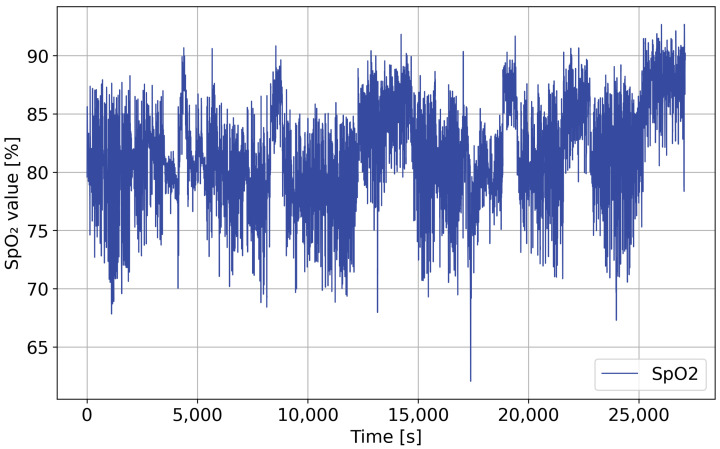

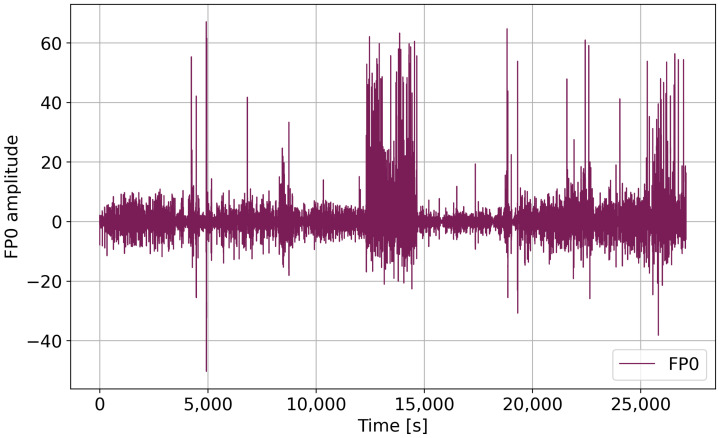
$SpO_{2}$ and FP0 signals after artifact removal and filtering.

After cleaning and filtering, both signals were divided into fixed-length windows covering short time intervals. Windowing allows the model to learn local temporal patterns such as apnea onset, airflow reduction, and the progression of oxygen decline. It also allows the dataset to be converted into multiple training samples per patient, improving model robustness. Figure 3 shows the $SpO_{2}$ and FP0 signals of one patient during the night after artifact and outlier removal.

### 3.3. Feature Engineering

To complement the raw physiological signals, a set of features was engineered to quantify specific aspects of abnormal breathing events. Desaturation events were identified in the $SpO_{2}$ waveform using thresholds related to amplitude drop and minimum event duration. Airflow cessation events were identified in the FP0 signal and paired with corresponding $SpO_{2}$ desaturations to ensure that extracted events were clinically meaningful. From each matched event pair, the following temporal and morphological parameters were calculated:

- t3–t1 mean: Defined as the average delay between the onset of FP0 cessation (t1) and the beginning of oxygen desaturation (t3). This parameter quantifies the latency between respiratory obstruction and its physiological manifestation in blood $SpO_{2}$.
- t4–t2 mean: Defined as the average delay between the resumption of FP0 (t2) and the start of oxygen recovery (t4). This reflects the time needed for $SpO_{2}$ to normalize once breathing resumes.
- delta_t_FP0_mean: The mean duration of FP0 cessation episodes, computed directly from the FP0 signal between markers t1 (start of apnea) and t2 (end of apnea). This value represents the average length of respiratory arrest events.
- $delta\_t\_SpO_{2}\_mean$: The mean duration of oxygen desaturation episodes, calculated as the time interval between t3 (start of desaturation) and t4 (end of desaturation). It provides a measure of how long $SpO_{2}$ remains depressed during events.
- $mean\_delta\_SpO_{2}$: The average desaturation difference, i.e., the difference between the initial $SpO_{2}$ value and the minimum value reached during all detected desaturation events throughout the night. This quantifies the drop in oxygen during the night.
- mean slope: The average slope of the desaturation curves, calculated as $\Delta SpO_{2}$/Δt during the fall phase of all events. It describes the rate of decline in $SpO_{2}$, distinguishing between abrupt and gradual desaturations.

In addition to the above signal-derived features, standard clinical parameters were included: age, gender (female: 1, male: 0), BMI, AHI, and heart rate. All features were then integrated into a feature matrix, where each row represents a patient and each column a clinical or derived feature, shown in Table 1. The additional parameters are shown in Table 2, along with their measurement units and clinical lower limits.

**Figure 4:**
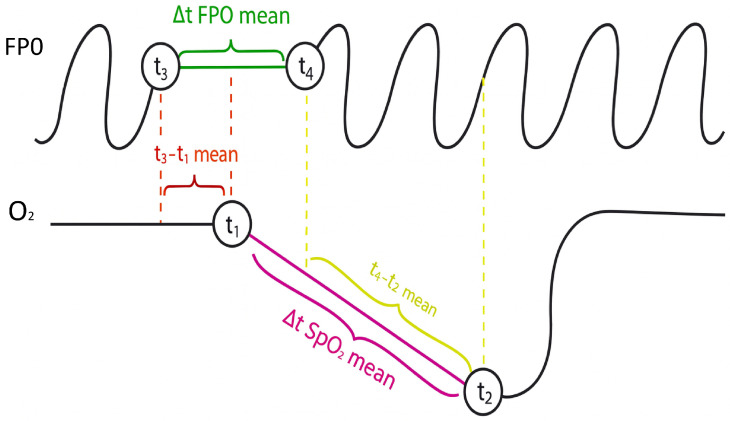
$SpO_{2}$ and FP0 signals with defined markers; illustration.

These features describe the physiological relationship between airflow changes and oxygen regulation, capturing clinically relevant respiratory patterns that may reflect underlying comorbidities Figure 4. All extracted features, along with demographic and clinical variables, were normalized to ensure consistent scaling across patients. The final dataset integrates three types of information: preprocessed $SpO_{2}$ and FP0 time series windows, computed temporal and morphological features, and clinical and demographic variables. This structure enables the DL model to simultaneously analyze short-term respiratory dynamics and longer-term patient characteristics.

### 3.4. Analysis of Data

To provide clinical context for the dataset, baseline characteristics were analyzed across patient subgroups defined by the presence of comorbidities (hypertension, diabetes mellitus, asthma/COPD), as well as an overall disease/no disease split. This analysis summarizes how demographic, clinical, and signal-derived parameters vary between groups and supports the interpretation of the extracted features used in subsequent modeling.

**Figure 5:**
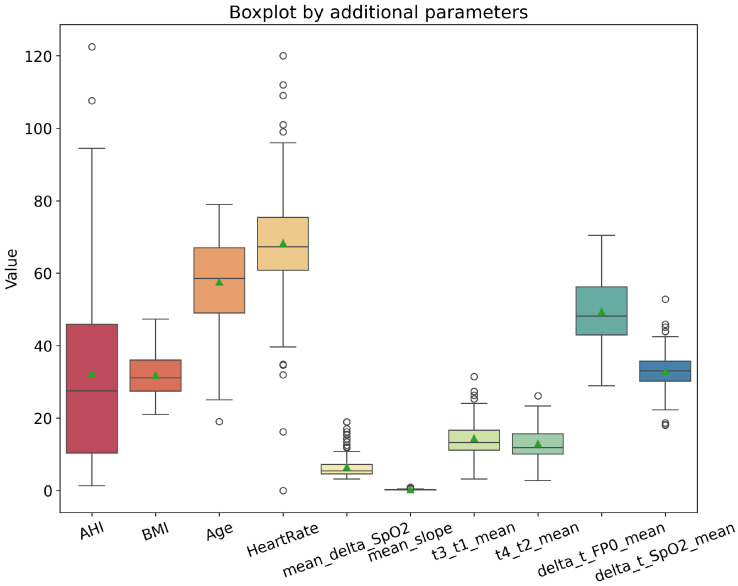
Box and whisker plots of selected clinical and signal-derived parameters used in the model. The central line indicates the median, the box the interquartile range (IQR), and whiskers extend to 1.5×IQR. Circles denote outliers, and triangles denote the mean.

Table 3 compares patients without comorbidities (NO: n=24) and those with at least one comorbidity (YES: n=120). For each comparison (no comorbidity vs. comorbidity and for each individual comorbidity subgroup), both p-values and an effect size were reported. In addition to p-values, effect sizes were reported using Cohen’s *d* (absolute values) [68], computed as the standardized difference between group means using the pooled standard deviation. Although small numerical differences are observed in several variables, BMI shows the most pronounced separation (p<0.01), with higher mean values in patients with comorbidities. In contrast, AHI values remain similar across groups. Figure 5 visualizes the distributions of selected parameters and illustrates substantial overlap between the two populations. Similar comparisons are reported for the individual comorbidity subgroups (Table 4 (YES: n=79), Table 5 (YES: n=65), and Table 6 (YES: n=65)), highlighting patterns associated with each diagnosis.

**Figure 6:**
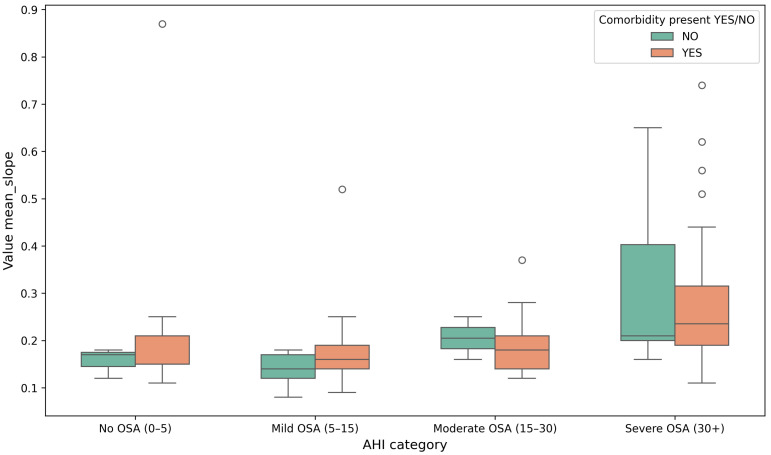
Distribution of the mean slope values across AHI categories for patients with and without comorbidities. Circles indicate outlier observations beyond the whiskers.

**Figure 7:**
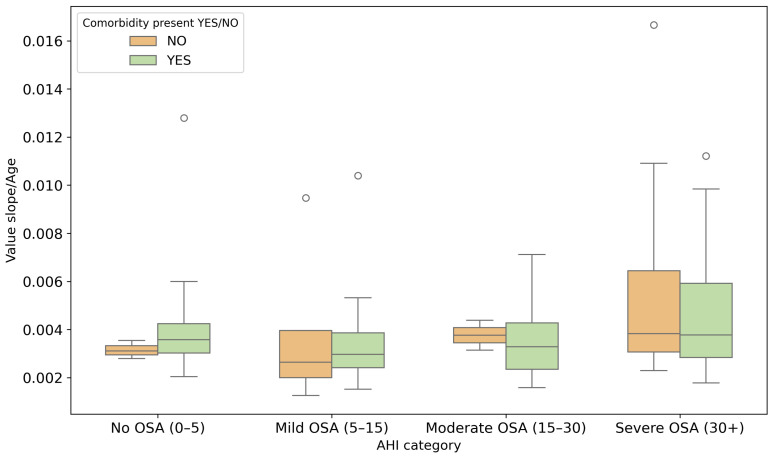
Distribution of slope-to-age ratio across AHI severity categories. Circles indicate outlier observations beyond the whiskers.

**Figure 8:**
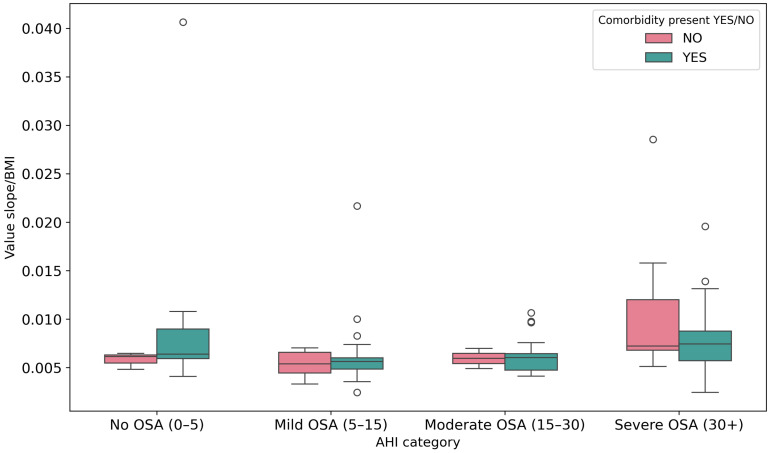
Distribution of slope-to-BMI ratio across AHI severity categories. Circles indicate outlier observations beyond the whiskers.

In addition to baseline comparisons, slope-based respiratory features were examined across AHI severity categories (Figure 6, Figure 7 and Figure 8). The mean slope increases with higher AHI levels, particularly in the severe OSA group, indicating greater variability in respiratory signal dynamics with increasing apnea severity. When normalized by age and BMI, the same trend remains visible, suggesting that respiratory slope characteristics capture meaningful changes in breathing morphology across severity strata. Statistical testing across severity categories showed increasing separation between patients with and without comorbidities, reaching significance in the moderate OSA group (p=0.03).

Overall, these observations support the subsequent modeling stage, in which both signal-derived features and clinical parameters are considered for comorbidity prediction. Even when univariate differences are modest, predictive information may arise from multivariate and nonlinear interactions that are not reflected in classical statistical comparisons.

### 3.5. Limitations of Data

Although the dataset provides valuable clinical information, several limitations should be acknowledged. The label distribution is imbalanced: hypertension is considerably more prevalent than diabetes mellitus and asthma/COPD, while multi-label combinations are rare. This imbalance increases the difficulty of multi-label learning, may bias the model toward the most frequent conditions, and can reduce the stability of performance estimates for underrepresented comorbidities. In addition, the dataset does not include healthy control subjects, since all individuals were referred for PSG due to suspected or confirmed sleep-disordered breathing, which may limit generalizability to screening or population-based settings.

Although full PSG contains multiple physiological channels, this study focused on two core channels [69] ($SpO_{2}$ and FP0) for model development. Full PSG recordings include additional modalities (e.g., EEG, ECG, EMG, respiratory belts) that provide complementary information about sleep stages, autonomic regulation, and respiratory effort and could potentially improve classification performance. Therefore, restricting the input to two channels reduces the available multimodal context and may limit the model’s ability to capture complex interactions between physiological systems. Finally, the dataset originates from a single clinical center and reflects local referral patterns, which may introduce selection bias and motivates further validation on independent cohorts.

## 4. Methods

### 4.1. Problem Definition

The goal of this study is to develop an MLC model capable of identifying three clinically relevant comorbidities commonly associated with OSA: hypertension, diabetes mellitus, and asthma/COPD. Each patient can simultaneously exhibit zero, one, two, or all three comorbidities. The predictive task therefore requires assigning a vector of three binary outputs, where each element indicates the presence or absence of a specific condition.

This setup differs from traditional single-label classification because labels are not mutually exclusive. The model must learn to capture shared patterns across conditions while also distinguishing features unique to each disease.

### 4.2. 1D-CNN Architecture

Convolutional Neural Networks (CNNs) are a core DL architecture originally developed for image analysis, where they extract spatial patterns using trainable filters [70]. Their fundamental mechanism for learning local features through convolution extends naturally to one-dimensional data, making them highly suitable for biomedical time series signals. In the context of sleep medicine, 1D-CNNs are effective because physiological waveforms such as $SpO_{2}$ and airflow contain characteristic temporal structures associated with respiratory instability and oxygen desaturation events. These temporal signatures can be difficult to capture using traditional ML methods but can be efficiently learned through convolutional layers that scan the signal and detect recurring patterns [71].

In 1D form, each convolutional filter slides along the temporal axis of the signal and computes a dot product between the kernel and local segments of the waveform. This enables the model to detect short-term events such as rapid desaturation declines or airflow cessations, as well as more gradual patterns related to apnea severity or recovery dynamics. Additional components such as activation functions, padding, and stride control the nonlinearity and temporal resolution of the learned representations. The use of the ReLU activation function enhances gradient flow and prevents saturation effects, while padding ensures that the temporal length of the output remains aligned with the input signal [72].

**Figure 9:**
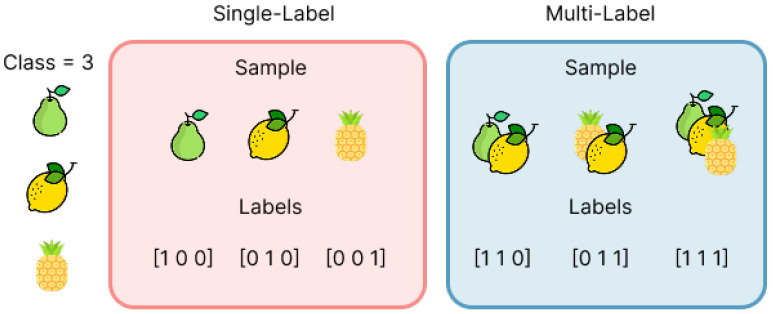
Example of single label vs. MLC.

The predictive task in this study is formulated as an MLC problem, where each patient may simultaneously exhibit several comorbidities rather than belonging to a single diagnostic category. This stands in contrast to traditional single-label classification, where each instance is associated with exactly one class. A simple visual illustration of this difference is shown in Figure 9, which compares mutually exclusive labels with multi-label assignments applicable to real-world biomedical data [73,74].

**Figure 10:**
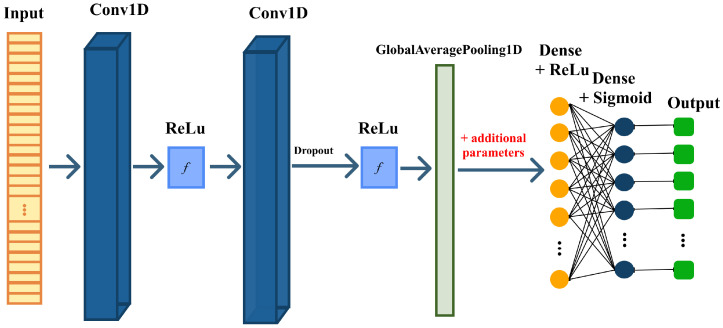
Conceptual overview of convolutional feature extraction in a CNN.

Within this learning framework, convolutional layers play a key role in extracting temporal patterns from biomedical signals. A conceptual overview of how convolutional operations progressively transform the input signal through stacked feature extraction blocks is shown in Figure 10, which illustrates the hierarchical flow from raw time series data to deeper learned representations [71].

Building on these concepts, the proposed model uses a multi-branch 1D-CNN architecture designed to integrate PSG time series with clinical information. Two branches process the physiological signals independently: one for oxygen saturation ($SpO_{2}$), one for the derivated $SpO_{2}$ signal and one for nasal airflow (FP0). Each branch contains convolutional layers that learn relevant temporal motifs, followed by batch normalization to stabilize training and dropout to reduce overfitting. Global Average Pooling (GAP) condenses each feature map into a compact representation, emphasizing dominant temporal patterns rather than specific signal positions. In parallel, a third branch processes clinical and signal-derived parameters such as age, BMI, AHI, heart rate, and respiratory timing features. This structured input is passed through a fully connected pathway to generate a dense embedding compatible with the signal-based representations. The outputs of all three branches are then concatenated to form a unified feature vector that captures temporal dependencies in the $SpO_{2}$ waveform, airflow-related respiratory patterns, and broader patient-level characteristics. To support multi-label prediction, the final output layer uses three independent sigmoid units, enabling simultaneous estimation of hypertension, diabetes mellitus, and asthma/COPD. This design aligns the model with the multi-label nature of the task.

**Figure 11:**
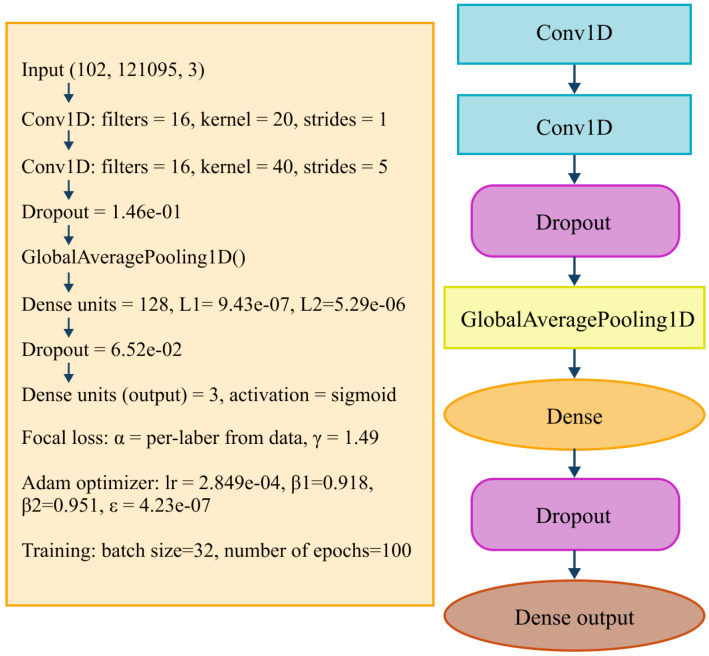
Multi-branch 1D-CNN architecture used for comorbidity prediction.

A detailed overview of the complete architecture, including convolutional hyperparameters, dense layers, dropout rates, and the focal loss configuration, is presented in Figure 11, which summarizes every component used in the final implementation.

Overall, the integration of convolutional feature extraction, clinical feature processing, and multi-label prediction enables the model to leverage diverse biomedical data and to learn both short-term physiological patterns and long-term patient characteristics. This architecture proved effective in identifying comorbidity-related signatures within PSG signals, supporting its use in automated risk assessment for OSA populations.

### 4.3. Class Imbalance Handling

The dataset exhibits notable class imbalance, especially for diabetes mellitus and asthma/COPD, which occur less frequently than hypertension. Multi-label combinations further amplify this imbalance and can bias the model toward majority classes. To address this issue, the training process incorporates weighted binary cross entropy, where each label is assigned a class-specific weight inversely proportional to its frequency. This ensures that rare comorbidity classes contribute more strongly to the loss function, encouraging the model to learn from underrepresented cases. A detailed analysis of comorbidity distribution was performed to calculate appropriate weights and to identify the imbalance between single-label, dual-label, and triple-label cases.

### 4.4. Evaluation Metrics

The evaluation of MLC models requires metrics that capture the fact that each instance may contain multiple labels simultaneously. Unlike single-label classification, where each sample has exactly one true class, MLC allows partial correctness, meaning that a prediction may overlap with the true label set even when the match is not exact. For this reason, model performance was assessed using a combination of label-based, example-based, threshold-independent, and error-based metrics [75,76].

Label-based metrics evaluate each comorbidity independently by computing standard binary measures such as accuracy, precision, recall, and F1-score [77]. Here, TP, FP, TN, and FN denote the number of true positives, false positives, true negatives, and false negatives, respectively, and *N* denotes the total number of samples. These metrics rely on the following definitions:

(1) $$Accuracy=\frac{TP+TN}{N}$$

(2) $$Precision=\frac{TP}{TP+FP}$$

(3) $$Recall=\frac{TP}{TP+FN}$$

(4) $$F1=\frac{2TP}{2TP+FP+FN}$$

To summarize results across all labels, macro and micro averaging were applied. Macro averaging assigns equal weight to each label, while micro averaging aggregates all true positives, false positives, true negatives, and false negatives across labels

(5) $$B_{macro}=\frac{1}{q}\sum_{j=1}^{q}B\left(TP_{j},FP_{j},TN_{j},FN_{j}\right)$$

(6) $$B_{micro}=B\left(\sum_{j=1}^{q}TP_{j},\;\sum_{j=1}^{q}FP_{j},\;\sum_{j=1}^{q}TN_{j},\;\sum_{j=1}^{q}FN_{j}\right)$$

Example-based metrics evaluate prediction correctness at the level of each patient. Subset accuracy is the strictest measure, requiring the entire predicted label set to match the true label set exactly,

(7) $$SubsetAccuracy=\frac{1}{N}\sum_{i=1}^{N}I\left(Y_{i}=Z_{i}\right)$$

Flat accuracy evaluates correctness at the level of individual labels,

(8) $$FlatAccuracy=\frac{1}{Nq}\sum_{i=1}^{N}\sum_{j=1}^{q}I\left(y_{ij}=z_{ij}\right)$$

Partial accuracy quantifies the overlap between the predicted and true label sets,

(9) $$PartialAccuracy=\frac{1}{N}\sum_{i=1}^{N}\frac{|Y_{i}\cap Z_{i}|}{|Y_{i}\cup Z_{i}|}$$

Threshold-independent metrics were also used. The area under the ROC curve (AUC-ROC) and the area under the precision–recall curve (AUC-PR) measure discriminative performance across different thresholds. AUC-PR is particularly informative for imbalanced datasets.

To quantify fine-grained prediction errors, Hamming loss was calculated,

(10) $$HammingLoss=\frac{1}{Nq}\sum_{i=1}^{N}\sum_{j=1}^{q}I\left(y_{ij}\neq z_{ij}\right)$$

Finally, model errors were analyzed using a multi-label confusion matrix (MLCM). Unlike traditional confusion matrices, which assume a single true class, the multi-label version computes true positives, false positives, true negatives, and false negatives separately for each comorbidity. This enables detailed inspection of label-wise misclassifications and reveals dependencies between comorbidities [78,79,80,81].

The combined use of these complementary metrics provides a comprehensive evaluation framework for assessing the models’ ability to detect multiple comorbidities simultaneously and supports a detailed interpretation of predictive performance.

## 5. Results

This section presents the quantitative evaluation of the proposed 1D-CNN architecture for MLC of comorbidities in patients with OSA. All experiments were conducted on a workstation equipped with an Intel® Core™ i5-1135G7 CPU (2.40 GHz) and 16 GB RAM. The complete training procedure for the final model required less than one hour, depending on the selected hyperparameters. At inference time, the model required only a few seconds per patient under the same hardware configuration.

The dataset was divided at the patient level into training (70%), validation (15%), and test (15%) subsets to prevent data leakage. Hyperparameter optimization was performed on the training and validation subsets, while the test subset was reserved exclusively for the final evaluation. The independent test set, fully unseen during model development, was used exclusively for the final evaluation. The model outputs three binary labels corresponding to hypertension, diabetes mellitus, and asthma/COPD.

Table 7 summarizes the raw MLCM, including class-wise precision and recall. The diagonal values represent correctly predicted labels, while non-diagonal entries represent misclassifications or clinically realistic co-occurrences.

Label $\lambda_{2}$ demonstrates perfect recall (1.00), indicating that all true instances were successfully detected, despite its relatively small prevalence. Labels $\lambda_{1}$ and $\lambda_{3}$ exhibit moderate recall (0.50 and 0.70), reflecting typical overlaps between clinically related comorbidities. To investigate cross-label relationships, the precision and recall matrices were computed from the raw MLCM.

High diagonal values in both tables confirm that the 1D-CNN maintains stable precision and recall across all labels. Misclassifications primarily occur among physiologically or clinically related comorbidities, which is expected due to natural co-occurrence patterns.

To address the study objective of evaluating multi-label comorbidity identification performance, we report both label-wise metrics derived from the multi-label confusion matrix and global multi-label evaluation scores on the independent test set. The proposed 1D-CNN demonstrates stable and generalizable performance across all metrics. The high precision for $\lambda_{1}$ and $\lambda_{3}$ (0.89 and 0.64) indicates a low false positive rate (Table 8), while $\lambda_{2}$ achieves extremely high recall (1.00), confirming the model’s ability to recognize all true cases of this comorbidity (Table 9). This behavior suggests that the network successfully differentiates subtle temporal features embedded in the $SpO_{2}$ and FP0 signals and effectively integrates structured clinical parameters.

Table 10 presents the full set of multi-label evaluation metrics. Subset accuracy (strictest measure) reached 0.286, indicating an exact match of all labels for 29% of samples. Flat and partial accuracy achieved markedly higher values (0.635), reflecting consistent partial correctness. F1-scores (macro, micro, and weighted) ranged between 0.53 and 0.55, demonstrating balanced performance across both frequent and rare labels. Macro AUC-ROC = 0.731 and AUC-PR = 0.750 indicate strong threshold-independent discriminative capability.

Moderate recall for $\lambda_{1}$ and $\lambda_{3}$ primarily stems from the natural overlap between comorbidities (e.g., hypertension and metabolic disorders), not from instability of the model. Importantly, such confusions are clinically plausible, as several of the considered conditions frequently co-occur in OSA patients and share overlapping physiological patterns. Precision and recall matrices confirm this by showing the highest off-diagonal confusion occurring between clinically correlated labels.

Global metrics further support model robustness. While subset accuracy is intentionally strict and penalizes any partially incorrect prediction, flat/partial accuracy better reflects practical screening utility by quantifying how many comorbidity labels are correctly identified per patient. The model achieves approximately two-thirds correctness at the label level (flat/partial accuracy = 0.635) and balanced F1-scores despite class imbalance. High macro AUC-ROC and AUC-PR values reflect excellent ranking performance even when labels overlap.

Overall, these results demonstrate that the CNN reliably extracts meaningful temporal patterns from physiological waveforms. Comorbidities exhibit consistent and interpretable prediction behavior, not random confusion. The architecture generalizes well to unseen patients, confirming robustness for real-world deployment.

To address the objective of assessing robustness across demographic subgroups, we performed a stratified evaluation by age, gender, and BMI on the independent test set. Furthermore, stratified analysis by age, gender, and BMI indicates that the model performs consistently across all demographic subsets.

**Figure 12:**
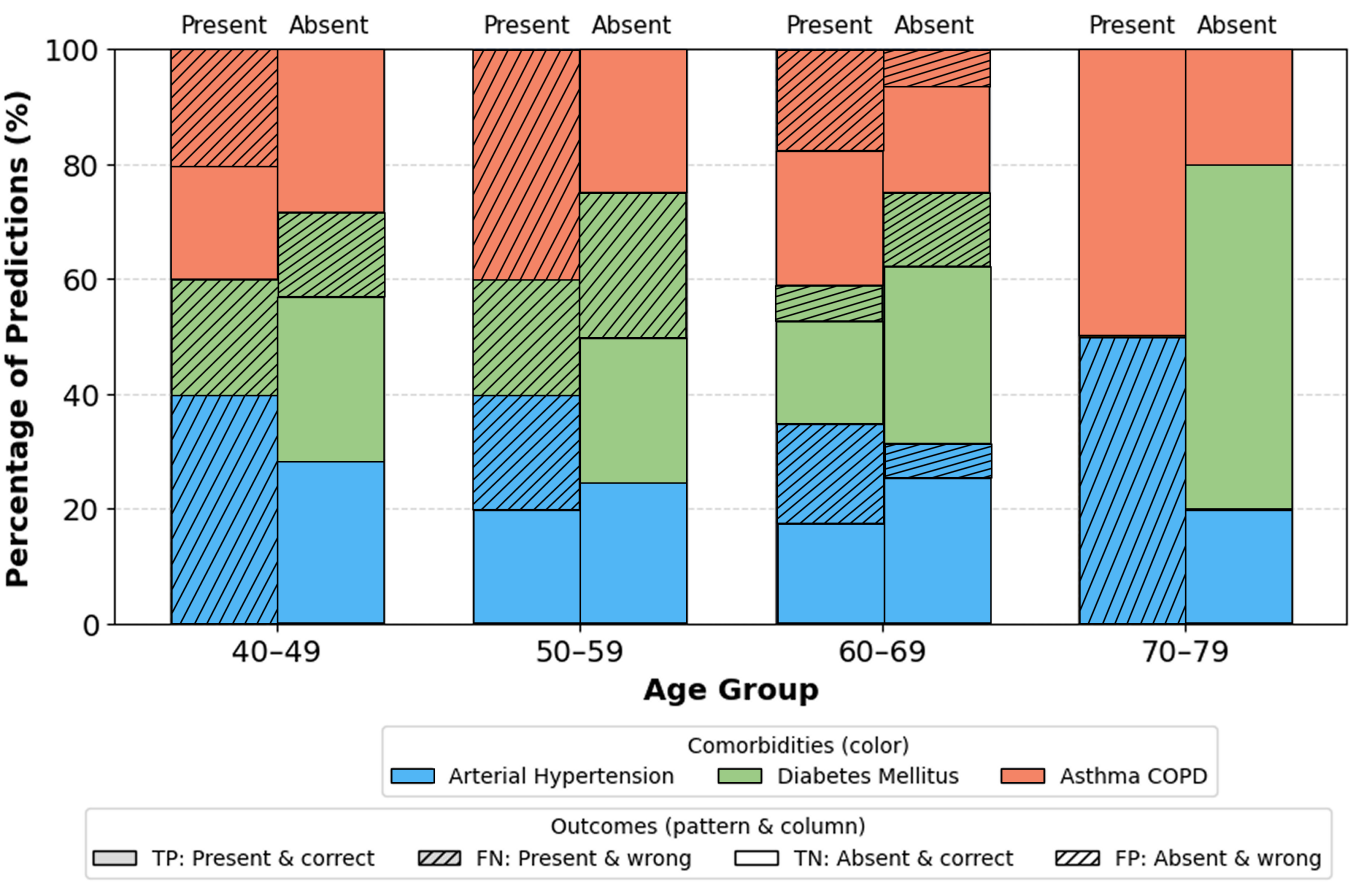
Correct and incorrect predictions per comorbidity by age group. Colors indicate comorbidity type (arterial hypertension, diabetes mellitus, asthma/COPD). For each age range, the left bar corresponds to comorbidity present and the right bar to absent; solid segments denote correct predictions (TP for present, TN for absent), and hatched segments denote incorrect predictions (FN for present, FP for absent).Abbreviations: TP, true positives; TN, true negatives; FP, false positives; FN, false negatives.

**Figure 13:**
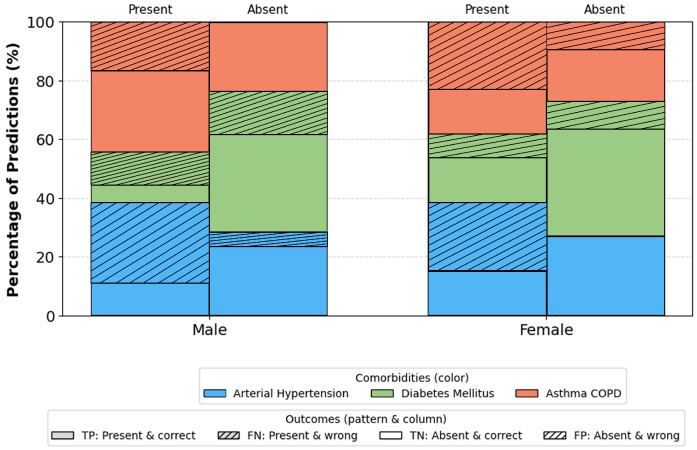
Correct and incorrect predictions per comorbidity by gender. Colors indicate comorbidity type (arterial hypertension, diabetes mellitus, asthma/COPD). For each gender, the left bar corresponds to comorbidity present and the right bar to absent; solid segments denote correct predictions (TP for present, TN for absent), and hatched segments denote incorrect predictions (FN for present, FP for absent). Abbreviations: TP, true positives; TN, true negatives; FP, false positives; FN, false negatives.

**Figure 14:**
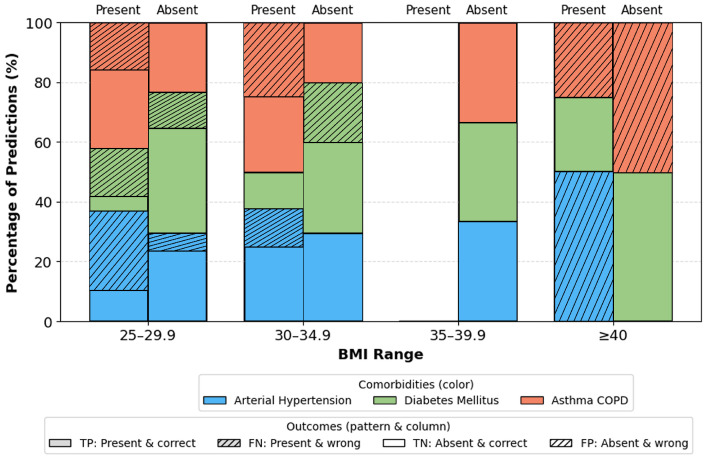
Correct and incorrect predictions per comorbidity by BMI range. Colors indicate comorbidity type (arterial hypertension, diabetes mellitus, asthma/COPD). For each BMI range, the left bar corresponds to comorbidity present and the right bar to absent; solid segments denote correct predictions (TP for present, TN for absent), and hatched segments denote incorrect predictions (FN for present, FP for absent). Abbreviations: TP, true positives; TN, true negatives; FP, false positives; FN, false negatives.

Age groups (Figure 12): Most stable performance occurs in the 40–69 range groups with the largest representation and typical comorbidity prevalence. Slight deviations in the youngest and oldest ranges are attributable to small sample sizes. Gender (Figure 13): No significant differences. Minor fluctuations reflect natural prevalence differences rather than model bias. BMI categories (Figure 14): Highest stability occurs in overweight/obese class I ranges (BMI 25–34.9). Extremes (BMI > 40) show a moderate increase in errors due to physiological variability and fewer samples.

Overall, the observed fluctuations at the extremes of the distributions are primarily attributable to smaller subgroup sample sizes rather than systematic model bias.

These findings indicate that performance variability is driven by dataset distribution rather than any inherent bias, suggesting suitability for diverse clinical populations.

The proposed 1D-CNN successfully captures short-term oxygen desaturation dynamics, airflow interruption patterns, and patient-level clinical characteristics. This multimodal integration enables reliable multi-label prediction of comorbidities directly from PSG-derived signals. Results confirm that $SpO_{2}$ and FP0 alone carry strong discriminative potential for identifying hypertension, diabetes mellitus, and asthma/COPD, supporting the development of cost-efficient screening tools. The strong threshold-independent performance (AUC-ROC, AUC-PR), balanced F1 scores, and clinically plausible confusion patterns highlight the models’ potential for real-world deployment.

### Analysis of the 1D-CNN Model and Comparison with Baseline Model Approach

Table 11 summarizes the MLC results of the proposed model and compares them with a baseline CNN model trained without additional clinical parameters. The baseline model used only the transformed time series signals as input, enabling a direct assessment of the contribution of the additional parameters to overall performance.

The proposed model achieves the best threshold-independent performance, with AUC-ROC (macro) = 0.731 and AUC-PR (macro) = 0.745. In contrast, the CNN baseline model, which uses only the transformed time series signals without additional clinical parameters, obtains substantially lower ranking performance (AUC-ROC = 0.459, AUC-PR = 0.599). This highlights the clear benefit of integrating additional clinical parameters alongside signal features in the proposed architecture, indicating that the proposed 1D-CNN ranks positive cases more reliably across labels and is better aligned with the underlying class imbalance. In threshold-dependent metrics, the CNN baseline also demonstrates inferior performance, with subset accuracy = 0.238 and Hamming loss = 0.413, while the proposed 1D-CNN achieves stronger performance, including Subset accuracy = 0.286 and Hamming loss = 0.365. Overall, the comparison confirms that predictive improvements stem not only from convolutional modeling but also from combining temporal features with clinically informative parameters.

## 6. Discussion

The overall objective of this study was to develop and evaluate a multimodal DL framework for multi-label identification of major comorbidities in patients with OSA by integrating $SpO_{2}$, FP0 airflow, and structured clinical parameters. The findings of this study demonstrate that the proposed multi-branch 1D-CNN model can effectively extract clinically meaningful temporal patterns from $SpO_{2}$ and FP0 signals and integrate them with structured clinical variables to identify key comorbidities associated with OSA. The model achieved balanced multi-label performance across hypertension, diabetes mellitus, and asthma/COPD, despite notable class imbalance within the dataset. This indicates that weighted loss functions and multimodal feature fusion successfully mitigated the dominance of majority labels and encouraged the network to learn discriminative representations even for less frequent comorbidities.

Recent literature increasingly supports the use of DL models for automated analysis of physiological signals in OSA screening, particularly when leveraging multimodal signal fusion to improve robustness and generalizability [82,83,84]. The raw MLCM (Table 7) shows that hypertension and asthma/COPD achieved moderate recall (0.50 and 0.70), while diabetes mellitus reached perfect recall (1.00). This result is particularly noteworthy given the relatively low prevalence of diabetes within the dataset, suggesting that the model learned subtle temporal clinical signatures specific to the metabolic profile of diabetic patients. Precision remained high across all labels (0.89–0.64), indicating low false positive rates and confirming that the classifier avoids overpredicting comorbidities, which is crucial for clinical usability.

Analysis of the precision and recall matrices (Table 8 and Table 9) further highlights that misclassifications predominantly occur between clinically related comorbidities, most notably hypertension and diabetes mellitus. This is consistent with well-known physiological and metabolic interactions in OSA patients, where sympathetic activation, intermittent hypoxia, and obesity contribute to overlapping risk profiles [85,86,87]. In addition, the bidirectional association between OSA and cardiometabolic dysfunction has been repeatedly highlighted in recent reviews and meta-analyses, supporting the clinical plausibility of these label overlaps [88]. These errors therefore likely reflect meaningful comorbidity co-occurrence rather than model instability.

Evaluation metrics (Table 10) reinforce these observations. Flat accuracy and partial accuracy of 0.635 show that approximately two-thirds of labels per patient were correctly predicted, while macro/micro/weighted F1-scores (0.533–0.551) indicate consistent performance across both frequent and rare labels. Threshold-independent metrics revealed strong discriminative capability (macro AUC-ROC = 0.731; AUC-PR = 0.750), confirming that the learned representations generalize well to unseen patient data. Combined, these results support the robustness of the proposed multimodal architecture. This aligns with recent methodological recommendations emphasizing threshold-independent evaluation (e.g., AUC-PR) for imbalanced clinical prediction tasks, where fixed threshold metrics may underestimate ranking performance [89,90].

Subgroup analyses, Figure 12, Figure 13 and Figure 14, provide additional insights into demographic generalization. The model maintains stable performance across gender, with only minor variations reflecting natural prevalence differences rather than systematic bias. Age-based performance shows the highest stability in the 40–69 cohort, consistent with the highest sample density, while extremes of age exhibit greater variance due to limited representation. Similarly, accuracy remains highest within BMI ranges 25–35, aligning with typical OSA and comorbidity prevalence, whereas reduced stability in BMI ≥ 40 groups reflects physiological heterogeneity and smaller sample sizes. These findings suggest that performance variability is driven primarily by data distribution rather than architectural limitations.

Importantly, the study demonstrates that rich comorbidity-related physiological information is encoded within only two PSG-derived signals, $SpO_{2}$ and FP0. The ability of the 1D-CNN to detect comorbidities without relying on full PSG channels (EEG, EMG, ECG) underscores the potential for simplified and more accessible diagnostic workflows. This aligns with the growing need for scalable, low-cost screening tools in clinical and home-based environments [90]. A comparable trend is observed in the broader OSA literature, where multimodal but reduced sensor approaches (e.g., oxygen saturation combined with other accessible signals) can achieve competitive performance while improving practicality and scalability [82].

While the majority of prior work focuses on OSA detection and severity estimation, studies addressing comorbidity level prediction remain limited, which makes direct comparison challenging and highlights the clinical novelty of the presented multi-label framework [84]. Additionally, some studies report that simpler or traditional ML approaches can appear competitive under fixed threshold metrics, particularly when probability calibration or per-label threshold tuning is not applied. This may partly explain why differences between models are sometimes smaller for subset/flat accuracy than for AUC-based metrics, despite clear improvements in ranking performance.

Overall, the results show that 1D-CNN-based multimodal learning offers a promising direction for early identification of OSA-related comorbidities. The model captures both short-term respiratory dynamics and long-term clinical characteristics, achieving clinically interpretable and stable performance. These findings support the future integration of such models into decision support systems and telemedicine platforms.

## 7. Limitations and Future Work

Despite the encouraging results, several limitations should be acknowledged. First, the dataset size (144 patients) is modest and originates from a single clinical center, which restricts the models’ exposure to broader population variability and may limit generalizability. Second, only two physiological channels ($SpO_{2}$ and FP0 nasal airflow) were used in this study. Although these signals are highly informative for capturing respiratory disturbances in OSA and contributed to strong predictive performance, the absence of additional PSG channels (e.g., EEG, ECG, EMG, and thoracoabdominal effort belts) reduces the physiological context available to the model. Future studies could incorporate a richer set of PSG modalities, supported by expert validation, to provide complementary information and potentially improve comorbidity classification. Future work should include external validation on datasets from other institutions and multi-center experiments to confirm generalizability across different clinical settings and patient populations.

Class imbalance remains another important challenge. Hypertension was substantially more prevalent than diabetes mellitus and asthma/COPD, while multi-label comorbidity combinations were rare, reflecting the underlying clinical distribution rather than a sampling artifact. Although weighted loss functions improved learning stability, performance variability across labels persisted, indicating the need for additional strategies such as data augmentation, synthetic minority oversampling (e.g., GAN-based generation), and targeted rebalancing techniques in future work.

Finally, model interpretability was not explicitly addressed. Although convolutional architectures can provide more structured feature extraction than fully connected models, the present study did not incorporate explainable AI (XAI) methods such as Grad-CAM, SHAP, or LIME. Integrating XAI techniques in future research could improve clinical transparency and trust by highlighting the signal segments and clinical variables that most strongly influence comorbidity predictions.

## 8. Conclusions

This study demonstrates that a multimodal 1D-CNN can integrate $SpO_{2}$ and FP0 airflow signals with structured clinical variables to identify multiple OSA-related comorbidities within a unified multi-label framework. The findings show that the proposed fusion-based approach can reliably detect hypertension, diabetes mellitus, and asthma/COPD using a reduced set of PSG-derived inputs, supporting the feasibility of comorbidity screening without relying on full polysomnography.

The subgroup analyses indicate stable performance across age, BMI, and gender strata, suggesting that the learned representations capture clinically meaningful physiological patterns rather than systematic demographic bias. These results highlight the potential of simplified multimodal architectures to support scalable risk assessment in both clinical and home monitoring scenarios.

Despite limitations related to the modest single-center cohort and residual label imbalance, the proposed framework provides a methodological basis for further development toward clinical translation. Future work should focus on external multi-center validation, expansion of datasets, incorporation of additional PSG modalities, and the integration of explainable AI techniques to improve transparency and clinical trust. Overall, the study supports the use of deep-learning-based analysis of simplified PSG signals as a promising direction for automated decision support tools aimed at early identification of comorbidity profiles in OSA patients.

## Figures and Tables

**Table 1:** Example of structured clinical and engineered features used as additional inputs to the proposed model alongside the $SpO_{2}$ and FP0 time series signals. Each row corresponds to one patient.

Age	Gender	BMI	AHI	HeartRate	t3_t1_mean	t4_t2_mean	delta_t_FP0_mean	delta_t_SpO2_mean	mean_delta_SpO2	mean_slope
58	1	29.30	2.2	90.0	7.82	7.04	38.76	32.22	4.70	0.18
63	1	44.59	77.2	72.0	20.71	14.14	37.73	18.16	9.99	0.62
68	0	33.95	122.5	90.0	16.72	13.04	38.83	18.70	7.20	0.45
68	0	29.05	72.0	58.0	13.26	10.42	47.03	30.94	7.15	0.24
54	0	29.01	18.7	62.0	8.43	9.73	45.42	36.03	5.04	0.15
...	...	...	...	...	...	...	...	...	...	...
61	1	37.00	35.1	81.4	12.85	11.85	49.09	28.38	4.87	0.21
48	0	45.00	39.9	79.5	11.55	9.60	50.50	36.75	4.10	0.11
70	0	38.00	61.3	39.6	23.70	15.56	57.59	38.40	18.98	0.42
64	0	31.60	41.8	67.2	9.77	12.35	48.95	29.53	4.33	0.16
66	1	30.00	33.9	57.8	6.87	8.03	46.79	35.63	6.43	0.19

**Table 2:** Additional physiological parameters used in the study with their measurement units and typical clinical ranges.

Parameter	Unit	Lower Bound (Clinical Minimum)
Age	years	18–90
Gender	female = 1, male = 0	0–1
BMI	kg/m^2^	18–60
AHI	events/hour	0–120
Heart rate	beats/min	40–120
$t_{3}-t_{1}$ mean	seconds	>3–5%
$t_{4}-t_{2}$ mean	seconds	>3–5%
$\Delta t_{\mathrm{FP}0}$ mean	seconds	≥10
$\Delta t_{{\mathrm{SpO}}_{2}}$ mean	seconds	≥10
Mean ΔSpO_2_	%	≥3%
Mean slope	%	≥0.02–0.05%

**Table 3:** Comparison of variables between patients without and with any comorbidity (NO: n=24, YES: n=120).

Additional Parameters	NO (Mean ± SD)	YES (Mean ± SD)	*p* Value	Effect Size (d)
Age	54.08 (±11.82)	58.33 (±10.89)	0.060	0.383
Gender [F = 1, M = 0]	0.38 (±0.48)	0.43 (±0.49)	0.327	0.102
BMI	29.19 (±4.47)	32.37 (±6.04)	0.003	0.545
AHI	32.15 (±27.66)	32.34 (±25.79)	0.488	0.007
Heart Rate	74.43 (±21.39)	67.09 (±14.44)	0.063	0.464
$t_{3}-t_{1}$ mean	15.67 (±5.65)	14.61 (±5.27)	0.207	0.198
$t_{4}-t_{2}$ mean	14.77 (±4.93)	13.07 (±4.78)	0.069	0.353
$\Delta t_{FP0}$ mean	51.73 (±8.77)	50.01 (±10.01)	0.204	0.175
$\Delta t_{SpO_{2}}$ mean	33.02 (±4.98)	32.96 (±5.25)	0.479	0.011
Mean ΔSpO_2_	6.93 (±4.11)	6.45 (±2.99)	0.296	0.150
Mean slope	0.24 (±0.15)	0.23 (±0.12)	0.381	0.080

Note: female = 1 (F), male = 0 (M). Effect size is reported as Cohen’s *d* (absolute values), computed using the pooled standard deviation.

**Table 4:** Comparison of variables between groups with and without hypertension (NO: n=65, YES: n=79).

Additional Parameters	NO (Mean ± SD)	YES (Mean ± SD)	*p* Value	Effect Size (d)
Age	53.96 (±10.95)	60.65 (±10.40)	0.000	0.626
Gender [F = 1, M = 0]	0.40 (±0.49)	0.43 (±0.50)	0.357	0.060
BMI	30.12 (±5.76)	33.26 (±5.70)	0.001	0.546
AHI	26.96 (±25.70)	36.70 (±25.63)	0.013	0.378
Heart Rate	70.52 (±17.25)	66.50 (±14.74)	0.072	0.252
t3_t1_mean	14.19 (±5.44)	15.29 (±5.22)	0.112	0.206
t4_t2_mean	12.89 (±4.84)	13.73 (±4.82)	0.151	0.173
delta_t_FP0_mean	49.70 (±8.82)	50.80 (±10.57)	0.248	0.112
delta_t_SpO_2__mean	32.75 (±4.80)	33.16 (±5.51)	0.320	0.079
mean_delta_SpO_2_	6.32 (±3.48)	6.70 (±2.95)	0.248	0.118
mean_slope	0.23 (±0.13)	0.24 (±0.13)	0.395	0.077

Note: female = 1 (F), male = 0 (M). Effect size is reported as Cohen’s *d* (absolute values), computed using the pooled standard deviation.

**Table 5:** Comparison of variables between groups with and without diabetes mellitus (NO: n=79, YES: n=65).

Additional Parameters	NO (Mean ± SD)	YES (Mean ± SD)	*p* Value	Effect Size (d)
Age	60.06 (±10.45)	55.62 (±11.32)	0.008	0.408
Gender [F = 1, M = 0]	0.35 (±0.48)	0.47 (±0.50)	0.083	0.244
BMI	33.22 (±6.08)	30.71 (±5.56)	0.006	0.428
AHI	36.85 (±27.21)	28.56 (±24.55)	0.031	0.317
Heart Rate	66.58 (±16.52)	69.74 (±15.50)	0.123	0.196
t3_t1_mean	14.23 (±5.08)	15.24 (±5.52)	0.133	0.190
t4_t2_mean	12.76 (±4.23)	13.84 (±5.25)	0.090	0.228
delta_t_FP0_mean	50.20 (±10.64)	50.38 (±9.11)	0.457	0.018
delta_t_SpO_2__mean	33.32 (±5.35)	32.69 (±5.06)	0.237	0.120
mean_delta_SpO_2_	6.63 (±2.74)	6.44 (±3.54)	0.359	0.061
mean_slope	0.24 (±0.13)	0.23 (±0.12)	0.357	0.079

Note: female = 1 (F), male = 0 (M). Effect size is reported as Cohen’s *d* (absolute values), computed using the pooled standard deviation.

**Table 6:** Comparison of variables between groups with and without asthma/COPD (NO: n=79, YES: n=65).

Additional Parameters	NO (Mean ± SD)	YES (Mean ± SD)	*p* Value	Effect Size (d)
Age	57.28 (±10.68)	57.91 (±11.53)	0.367	0.057
Gender [F = 1, M = 0]	0.46 (±0.50)	0.38 (±0.49)	0.163	0.161
BMI	31.80 (±6.02)	31.88 (±5.85)	0.466	0.013
AHI	29.78 (±24.21)	34.38 (±27.41)	0.145	0.178
Heart Rate	68.21 (±12.20)	68.40 (±18.62)	0.472	0.012
t3_t1_mean	14.90 (±5.51)	14.70 (±5.21)	0.416	0.037
t4_t2_mean	13.27 (±5.18)	13.42 (±4.55)	0.427	0.030
delta_t_FP0_mean	49.98 (±9.60)	50.57 (±10.01)	0.361	0.060
delta_t_SpO_2__mean	32.65 (±5.17)	33.24 (±5.22)	0.249	0.113
mean_delta_SpO_2_	6.19 (±3.19)	6.81 (±3.19)	0.126	0.194
mean_slope	0.23 (±0.12)	0.24 (±0.13)	0.234	0.080

Note: female = 1 (F), male = 0 (M). Effect size is reported as Cohen’s *d* (absolute values), computed using the pooled standard deviation.

**Table 7:** Raw MLCM with class-wise recall and precision. Abbreviations: GT, ground truth; MLCM, multi-label confusion matrix; $\lambda_{1}$, hypertension; $\lambda_{2}$, diabetes mellitus; $\lambda_{3}$, asthma/COPD.

GT	$\lambda_{1}$	$\lambda_{2}$	$\lambda_{3}$	Recall
$\lambda_{1}$	4.0	0.0	4.0	0.50
$\lambda_{2}$	0.0	3.0	0.0	1.00
$\lambda_{3}$	0.5	2.5	7.0	0.70
**Precision**	0.89	0.55	0.64	

**Table 8:** Precision matrix (column normalized) derived from the raw MLCM. Abbreviations: $\lambda_{1}$, hypertension; $\lambda_{2}$, diabetes mellitus; $\lambda_{3}$, asthma/COPD.

	$\lambda_{1}$	$\lambda_{2}$	$\lambda_{3}$
$\lambda_{1}$	0.89	0.00	0.36
$\lambda_{2}$	0.00	0.55	0.00
$\lambda_{3}$	0.11	0.45	0.64

**Table 9:** Recall matrix (row normalized) derived from the raw MLCM. Abbreviations: $\lambda_{1}$, hypertension; $\lambda_{2}$, diabetes mellitus; $\lambda_{3}$, asthma/COPD.

	$\lambda_{1}$	$\lambda_{2}$	$\lambda_{3}$
$\lambda_{1}$	0.50	0.00	0.50
$\lambda_{2}$	0.00	1.00	0.00
$\lambda_{3}$	0.05	0.25	0.70

**Table 10:** Summary of evaluation metrics. Abbreviations: AUC-ROC, area under the receiver operating characteristic curve; AUC-PR, area under the precision–recall curve; TP, true positives; FP, false positives; FN, false negatives; TN, true negatives.

Metric	Value	Definition	Interpretation
Subset accuracy	0.286	Exact match across all labels per sample.	Strictest metric, a sample counts as correct only if *all* labels are predicted correctly. Lower values are expected in multi-label tasks with partial hits.
Flat accuracy	0.635	Bit-level accuracy over all labels (flattened).	Indicates frequent partial correctness at the label (bit) level across samples.
Partial accuracy	0.635	Mean per sample label match ratio.	Aligns with flat accuracy, showing that, on average, about two-thirds of labels per sample are correct.
$B_{\mathrm{macro}}$ (F1-macro)	0.533	Unweighted mean F1 across labels.	Treats rare and frequent labels equally, lower due to class imbalance and harder labels.
$B_{\mathrm{micro}}$ (F1-micro)	0.549	Global F1 aggregating TP/FP/FN over all labels.	Reflects overall balance of precision/recall across the dataset.
$B_{\mathrm{weighted}}$ (F1-weighted)	0.551	F1 weighted by label frequency.	Slight uplift vs. macro due to dominance of frequent labels.
AUC-ROC (macro)	0.731	Mean area under ROC curve across labels (one vs. rest).	Threshold independent discrimination, higher indicates better ranking of positives vs. negatives across labels.
AUC-PR (macro)	0.750	Mean area under precision–recall curve across labels.	More informative under class imbalance, higher indicates better detection of positives with fewer false alarms.
Hamming loss	0.365	Fraction of misclassified labels over all samples and labels.	Complement of flat accuracy (here ≈1−0.635); lower is better.

**Table 11:** Comparison of multi-label classifiers on the test set.

Model	Subset Acc	Flat Acc	Partial Acc	F1- Macro	F1- Micro	F1- Weighted	AUC-ROC (Macro)	AUC-PR (Macro)	Hamming Loss
CNN (proposed)	0.286	0.635	0.635	0.533	0.549	0.551	0.731	0.750	0.365
CNN (baseline)	0.143	0.508	0.508	0.348	0.392	0.318	0.459	0.599	0.492

Note: Subset accuracy denotes exact match accuracy (all labels correct). Flat accuracy represents overall label-wise accuracy, while partial accuracy denotes average per-sample label accuracy. Macro/micro/weighted F1 are reported for MLC. AUC-ROC and AUC-PR are macro averaged across labels. Lower Hamming loss indicates fewer label-wise classification errors.

## Data Availability

The PSG recordings and associated clinical data were de-identified prior to analysis and handled in compliance with applicable data protection regulations. Due to patient privacy and ethical restrictions, the raw data are not publicly available.

## References

[1] Benjafield A.V., Ayas N.T., Eastwood P.R., Heinzer R., Ip M.S.M., Morrell M.J., Nunez C.M., Patel S.R., Penzel T., Pépin J. (2019). Estimation of the global prevalence and burden of obstructive sleep apnoea: A literature-based analysis. Lancet Respir. Med..

[2] Iannella G., Pace A., Bellizzi M.G., Magliulo G., Greco A., De Virgilio A., Croce E., Gioacchini F.M., Re M., Costantino A. (2025). The Global Burden of Obstructive Sleep Apnea. Diagnostics.

[3] Abrishami A., Khajehdehi A., Chung F. (2010). A systematic review of screening questionnaires for obstructive sleep apnea. Can. J. Anesth..

[4] Deviaene M., Varon C., Testelmans D., Buyse B., Van Huffel S. (2017). Assessing cardiovascular comorbidities in sleep apnea patients using SpO_2_. Proceedings of the 2017 Computing in Cardiology (CinC).

[5] Chadia K., Archontogeorgis K., Drakopanagiotakis F., Bonelis K., Anevlavis S., Steiropoulos P. (2025). Clinical and Sleep Characteristics and the Effect of CPAP Treatment on Obese Patients with Obstructive Sleep Apnea and Asthma—A Retrospective Study. Healthcare.

[6] Gentile S., Monda V.M., Guarino G., Satta E., Chiarello M., Caccavale G., Mattera E., Marfella R., Strollo F. (2025). Obstructive Sleep Apnea and Type 2 Diabetes: An Update. J. Clin. Med..

[7] Tondo P., Hoxhallari A., Lacedonia D., Magaletti P., Sabato R., Foschino Barbaro M.P., Scioscia G. (2024). The CORE syndrome: An overlap of severe asthma, obstructive sleep apnea, rhinosinusitis, and esophageal reflux. Sleep Breath..

[8] Kainulainen S., Töyräs J., Oksenberg A., Korkalainen H., Sefa S., Kulkas A., Leppänen T. (2019). Severity of desaturations reflects OSA-related daytime sleepiness better than AHI. J. Clin. Sleep Med..

[9] Demko B.G. (2018). The Evolution of Oral Appliance Therapy for Snoring and Sleep Apnea: Where Did We Come From, Where Are We, and Where Are We Going?. Sleep Med. Clin..

[10] Marin J.M., Carrizo S.J., Vicente E., Agusti A.G. (2005). Long-term cardiovascular outcomes in men with obstructive sleep apnoea-hypopnoea with or without treatment with continuous positive airway pressure: An observational study. Lancet.

[11] Bahammam A., Gacuan D., George S., Acosta K.L., Pandi-Perumal S.R., Gupta R. (2016). Polysomnography I: Procedure and Technology. Synopsis of Sleep Medicine.

[12] Gottlieb D.J., Punjabi N.M. (2020). Diagnosis and management of obstructive sleep apnea: A review. JAMA.

[13] Slowik J.M., Sankari A., Collen J.F. (2025). Obstructive Sleep Apnea. StatPearls.

[14] Lipton Z., Kale D., Elkan C., Wetzel R. (2015). Learning to Diagnose with LSTM Recurrent Neural Networks. arXiv.

[15] Pattipati M., Gudavalli G., Zin M., Dhulipalla L., Kolack E., Karki M., Devarakonda P.K., Yoe L. (2022). Continuous Positive Airway Pressure vs Mandibular Advancement Devices in the Treatment of Obstructive Sleep Apnea: An Updated Systematic Review and Meta-Analysis. Cureus.

[16] Hussein O., Alkhader A., Gohar A., Bhat A. (2024). Home Sleep Apnea Testing for Obstructive Sleep Apnea. Mo. Med..

[17] Espinosa M.A., Ponce P., Molina A., Borja V., Torres M.G., Rojas M. (2023). Advancements in Home-Based Devices for Detecting Obstructive Sleep Apnea: A Comprehensive Study. Sensors.

[18] Zovko K., Šerić L., Perković T., Belani H., Šolić P. (2023). IoT and health monitoring wearable devices as enabling technologies for sustainable enhancement of life quality in smart environments. J. Clean. Prod..

[19] Hüsken M., Stagge P. (2003). Recurrent neural networks for time series classification. Neurocomputing.

[20] Afsa I., Ansari M.Y., Paul S., Halabi O., Alataresh E., Shah J., Hamze A., Aboumarzouk O., Al-Ansari A., Dakua S.P. (2025). Development and Validation of a Class Imbalance-Resilient Cardiac Arrest Prediction Framework Incorporating Multiscale Aggregation, ICA and Explainability. IEEE Trans. Biomed. Eng..

[21] Liu S., Fu B., Wang W., Liu M., Sun X. (2022). Dynamic Sepsis Prediction for Intensive Care Unit Patients Using XGBoost-Based Model With Novel Time-Dependent Features. IEEE J. Biomed. Health Inform..

[22] Kim Y., Koo J., Lee S., Song H., Lee M. (2023). Explainable AI Warning Model Using Ensemble Approach for In-Hospital Cardiac Arrest Prediction: A Retrospective Cohort Study. J. Med. Internet Res..

[23] Kobir M., Machado P., Lotfi A., Haider D., Ihianle I. (2025). Enhancing Multi-User Activity Recognition in an Indoor Environment with Augmented Wi-Fi Channel State Information and Transformer Architectures. Sensors.

[24] Hossen M.K., Peng Y.T., Shao A., Chen M. (2025). An ODE based neural network approach for PM2.5 forecasting. Sci. Rep..

[25] Xie J., Wang Z., Yu Z., Ding Y., Guo B. (2024). Prototype Learning for Medical Time Series Classification via Human–Machine Collaboration. Sensors.

[26] Sirrianni J., Sezgin E., Claman D., Linwood S. (2022). Medical Text Prediction and Suggestion Using Generative Pretrained Transformer Models with Dental Medical Notes. Methods Inf. Med..

[27] Tian M., Chen B., Guo A., Jiang S., Zhang A. (2024). Reliable generation of privacy-preserving synthetic electronic health record time series via diffusion models. J. Am. Med Inform. Assoc. JAMIA.

[28] Huo Z., Booth J., Monks T., Knight P., Watson L., Peters M., Pagel C., Ramnarayan P., Li K. (2025). Dynamic mortality prediction in critically Ill children during interhospital transports to PICUs using explainable AI. Npj Digit. Med..

[29] Lin P.K., Chiu Y.H., Huang C.J., Wang C.Y., Pan M.L., Wang D.W., Liao H.Y., Chen Y.S., Kuan C.H., Lin S.Y. (2022). PADAr: Physician-oriented artificial intelligence-facilitating diagnosis aid for retinal diseases. J. Med Imaging.

[30] Sharmila V.J., Jemi F.D. (2021). Deep Learning Algorithm for COVID-19 Classification Using Chest X-Ray Images. Comput. Math. Methods Med..

[31] Alp S., Akan-R.Farshi T., Bhuiyan M.S., Disbrow E., Conrad S., Vanchiere J., Kevil C., Bhuiyan M.A.N. (2024). Joint transformer architecture in brain 3D MRI classification: Its application in Alzheimer’s disease classification. Sci. Rep..

[32] Ahmad S., Ahmad Z., Kim J.M. (2022). A Centrifugal Pump Fault Diagnosis Framework Based on Supervised Contrastive Learning. Sensors.

[33] Rahaman Wahab Sait A., Alkhurayyif Y. (2025). Lightweight hybrid transformers-based dyslexia detection using cross-modality data. Sci. Rep..

[34] Jovic A., Frid N., Brkic K., Cifrek M. (2025). Interpretability and accuracy of machine learning algorithms for biomedical time series analysis—A scoping review. Biomed. Signal Process. Control.

[35] Saikumar K., Rajesh V., Srivastava G., Lin J.C.W. (2022). Heart disease detection based on internet of things data using linear quadratic discriminant analysis and a deep graph convolutional neural network. Front. Comput. Neurosci..

[36] Heydarian M., Doyle T.E., Samavi R. (2022). MLCM: Multi-Label Confusion Matrix. IEEE Access.

[37] Kimura A., Mitsukura Y., Oya A., Matsumoto M., Nakamura M., Kanaji A., Miyamoto T. (2021). Objective characterization of hip pain levels during walking by combining quantitative electroencephalography with machine learning. Sci. Rep..

[38] Patil S., Kukreja D. (2025). Deep Reinforced Cognitive Analytics Algorithm (DRCAM): An Advanced Method to early detection of Cognitive skill impairment using Deep Learning and Reinforcement Learning. MethodsX.

[39] Khan A., Basit M.S., Farooq O., Khan Y.U., Shameem M. (2023). Mitigating the class imbalance effect in Sleep Apnea Classification. Proceedings of the 2023 International Conference on Recent Advances in Electrical, Electronics & Digital Healthcare Technologies (REEDCON).

[40] Sheta A., Turabieh H., Thaher T., Too J., Mafarja M., Hossain M.S., Surani S.R. (2021). Diagnosis of Obstructive Sleep Apnea from ECG Signals Using Machine Learning and Deep Learning Classifiers. Appl. Sci..

[41] Kumar C.B., Bhongade A., Gandhi T.K., Panigrahi B.K. (2023). Recognition of Obstructive Sleep Apnea from a Single Lead Electrocardiogram Signal Using a ResNet Model. Proceedings of the 2023 14th International Conference on Computing Communication and Networking Technologies (ICCCNT).

[42] Kumar C.B., Mondal A.K., Bhatia M., Panigrahi B.K., Gandhi T.K. (2023). Self-Supervised Representation Learning-Based OSA Detection Method Using Single-Channel ECG Signals. IEEE Trans. Instrum. Meas..

[43] Zhang Y., Shi Y., Su Y., Cao Z., Li C., Xie Y., Niu X., Yuan Y., Ma L., Zhu S. (2025). Detection and severity assessment of obstructive sleep apnea according to deep learning of single-lead electrocardiogram signals. J. Sleep Res..

[44] Khan A., Biswas S.K., Chunka C. (2025). An Optimized Obstructive Sleep Apnea Detection Model Using Particle Swarm Optimization and Machine Learning. Proceedings of the 2025 10th IEEE International Conference on Integrated Circuits, Design, and Verification (ICDV).

[45] Khan A., Biswas S.K., Chunka C., Barman S. (2024). Ensembled Obstructive Sleep Apnea Detection Using Extra Tree Ensemble Technique. Proceedings of the 2024 2nd International Conference on Advancement in Computation & Computer Technologies (InCACCT).

[46] Satapathy S.K., Pattnaik S., Rath R. (2022). Automated Sleep Staging Classification System Based On Convolutional Neural Network Using Polysomnography Signals. Proceedings of the 2022 IEEE Delhi Section Conference (DELCON).

[47] Jain S., Singhal A., Rai J.K., Sharma P. (2025). Polysomnography based Sleep Stage Classification using Deep Learning for Sleep Apnea Pediatric Subjects. Proceedings of the 2025 4th OPJU International Technology Conference (OTCON) on Smart Computing for Innovation and Advancement in Industry 5.0.

[48] Shalini M., Gagan K.M., Dillon D.P., Arshad Irfan S., Sasirekha S.P., Mekala N. (2025). SomnoDx: A Real-Time Deep Learning Framework for Multimodal Sleep Disorder Diagnosis. Proceedings of the 2025 3rd International Conference on Self Sustainable Artificial Intelligence Systems (ICSSAS).

[49] Jirakittayakorn N., Manupibul U., Wongsawat Y., Mitrirattanakul S. (2024). RespNet: A Dual-Network Approach for Automated OSA Severity Classification Utilizing PSG Type III Signals. IEEE Access.

[50] Hanif U., Leary E., Schneider L., Paulsen R., Morse A., Blackman A., Schweitzer P., Kushida C., Liu S., Jennum P. (2021). Estimation of Apnea-Hypopnea Index Using Deep Learning On 3-D Craniofacial Scans. IEEE J. Biomed. Health Inform..

[51] He Z., Xiao Y., Wu X., Liang Y., Zhou Y., An G. (2023). An Automatic Assessment Model of Adenoid Hypertrophy in MRI Images Based on Deep Convolutional Neural Networks. IEEE Access.

[52] Qiu X., Wang C., Li B., Tong H., Tan X., Yang L., Tao J., Huang J. (2024). An audio-semantic multimodal model for automatic obstructive sleep Apnea-Hypopnea Syndrome classification via multi-feature analysis of snoring sounds. Front. Neurosci..

[53] Singh S., Anisi M.H., Jindal A., Jarchi D. (2024). Smart Multimodal In-Bed Pose Estimation Framework Incorporating Generative Adversarial Neural Network. IEEE J. Biomed. Health Inform..

[54] Kuan Y.C., Hong C.T., Chen P.C., Liu W.T., Chung C.C. (2022). Logistic regression and artificial neural network-based simple predicting models for obstructive sleep apnea by age, sex, and body mass index. Math. Biosci. Eng. MBE.

[55] Javeed A., Berglund J.S., Dallora A.L., Saleem M.A., Anderberg P. (2023). Predictive Power of XGBoost BiLSTM Model: A Machine Learning Approach for Accurate Sleep Apnea Detection Using Electronic Health Data. Int. J. Comput. Intell. Syst..

[56] Kim Y.J., Jeon J.S., Cho S.E., Kim K.G., Kang S.G. (2021). Prediction Models for Obstructive Sleep Apnea in Korean Adults Using Machine Learning Techniques. Diagnostics.

[57] Troncoso-García A.R., Martínez-Ballesteros M., Martínez-Álvarez F., Lora A.T. (2022). Explainable machine learning for sleep apnea prediction. Procedia Comput. Sci..

[58] Shi Y., Ma L., Chen X., Li W., Feng Y., Zhang Y., Cao Z., Yuan Y., Xie Y., Liu H. (2022). Prediction model of obstructive sleep apnea-related hypertension: Machine learning-based development and interpretation study. Front. Cardiovasc. Med..

[59] Li E., Ai F., Liang C. (2024). A machine learning model to predict the risk of depression in US adults with obstructive sleep apnea hypopnea syndrome: A cross-sectional study. Front. Public Health.

[60] Xu Y., Duan S., Yin W., Yang Y., Zhang X., Wang J. (2025). Machine learning-enhanced prediction model for atrial fibrillation development in patients with concurrent type 2 diabetes and obstructive sleep apnea syndrome: A comorbidity perspective. Nutr. Metab..

[61] Li A., Roveda J.M., Powers L.S., Quan S.F. (2022). Obstructive sleep apnea predicts 10-year cardiovascular disease-related mortality in the Sleep Heart Health Study: A machine learning approach. J. Clin. Sleep Med..

[62] Korompili G., Amfilochiou A., Kokkalas L., Mitilineos S.A., Tatlas N.A., Kouvaras M., Kastanakis E., Maniou C., Potirakis S.M. (2021). PSG-Audio, a scored polysomnography dataset with simultaneous audio recordings for sleep apnea studies. Sci. Data.

[63] Philips Healthcare (2024). Alice 6 LDe Diagnostic Sleep System. https://www.philips.com.bh/healthcare/product/HC1063311/alice-6-lde-diagnostic-sleep-system.

[64] Wikimedia Commons Sleep. https://commons.wikimedia.org/wiki/File:Sleep_studies.jpg.

[65] VanderPlas J. (2016). Python Data Science Handbook: Essential Tools for Working with Data. Python Data Science Handbook: Essential Tools for Working with Data.

[66] Crenna F., Rossi G.B., Berardengo M. (2021). Filtering Biomechanical Signals in Movement Analysis. Sensors.

[67] Kawala-Sterniuk A., Podpora M., Pelc M., Blaszczyszyn M., Gorzelanczyk E.J., Martinek R., Ozana S. (2020). Comparison of Smoothing Filters in Analysis of EEG Data for the Medical Diagnostics Purposes. Sensors.

[68] Larner A.J. (2014). Effect Size (Cohen’s d) of Cognitive Screening Instruments Examined in Pragmatic Diagnostic Accuracy Studies. Dement. Geriatr. Cogn. Disord. Extra.

[69] Levendowski D.J., Hamilton G.S., St. Louis E.K., Penzel T., Dawson D., Westbrook P.R. (2019). A comparison between auto-scored apnea-hypopnea index and oxygen desaturation index in the characterization of positional obstructive sleep apnea. Nat. Sci. Sleep.

[70] Kelleher J.D. (2019). Deep Learning.

[71] Kim P. (2017). Matlab deep learning. Mach. Learn. Neural Netw. Artif. Intell..

[72] Albawi S., Mohammed T.A., Al-Zawi S. (2017). Understanding of a convolutional neural network. Proceedings of the 2017 International Conference on Engineering and Technology (ICET).

[73] Gibaja E., Ventura S. (2014). Multi-label learning: A review of the state of the art and ongoing research. Wiley Interdiscip. Rev. Data Min. Knowl. Discov..

[74] Koyejo O.O., Natarajan N., Ravikumar P.K., Dhillon I.S. (2015). Consistent multilabel classification. Adv. Neural Inf. Process. Syst..

[75] Du J., Chen Q., Peng Y., Xiang Y., Tao C., Lu Z. (2019). ML-Net: Multi-label classification of biomedical texts with deep neural networks. J. Am. Med Inform. Assoc. JAMIA.

[76] Pereira R.B., Plastino A., Zadrozny B., Merschmann L.H. (2018). Correlation analysis of performance measures for multi-label classification. Inf. Process. Manag..

[77] Krstinić D., Braović M., Šerić L., Božić-Štulić D. (2020). Multi-label classifier performance evaluation with confusion matrix. Comput. Sci. Inf. Technol..

[78] Koço S., Capponi C. (2013). On multi-class classification through the minimization of the confusion matrix norm. Proceedings of the Asian Conference on Machine Learning.

[79] Canbek G., Sagiroglu S., Temizel T.T., Baykal N. (2017). Binary classification performance measures/metrics: A comprehensive visualized roadmap to gain new insights. Proceedings of the 2017 International Conference on Computer Science and Engineering (UBMK).

[80] Labatut V., Cherifi H. (2011). Evaluation of performance measures for classifiers comparison. arXiv.

[81] Fawcett T. (2006). An introduction to ROC analysis. Pattern Recognit. Lett..

[82] Zhang W., Li X., Chen M., Wang R. (2025). Construction and validation of a machine learning-based risk prediction model for sleep quality in patients with obstructive sleep apnea. J. Sleep Res..

[83] Hemrajani P., Dhaka V.S., Rani G., Verma S., Kavita, Wozniak M., Shafi J., Ijaz M.F. (2025). Integrating physiological signals for enhanced sleep apnea diagnosis with SleepNet. Sci. Rep..

[84] Bazoukis G., Bollepalli S.C., Chung C.T., Li X., Tse G., Bartley B.L., Batool-Anwar S., Quan S.F., Armoundas A.A. (2023). Application of artificial intelligence in the diagnosis of sleep apnea. J. Clin. Sleep Med..

[85] Shiina K. (2024). Obstructive sleep apnea-related hypertension: A review of the literature and clinical management strategy. Hypertens. Res..

[86] Javaheri S., Javaheri S., Somers V.K., Gozal D., Mokhlesi B., Mehra R., McNicholas W.T., Zee P.C., Campos-Rodriguez F., Martinez-Garcia M.A. (2024). Interactions of Obstructive Sleep Apnea With the Pathophysiology of Cardiovascular Disease, Part 1: JACC State-of-the-Art Review. J. Am. Coll. Cardiol..

[87] Maniaci A., Lavalle S., Parisi F.M., Barbanti M., Cocuzza S., Iannella G., Magliulo G., Pace A., Lentini M., Masiello E. (2024). Impact of Obstructive Sleep Apnea and Sympathetic Nervous System on Cardiac Health: A Comprehensive Review. J. Cardiovasc. Dev. Dis..

[88] Huang H., Chen Z. (2025). Association between obstructive sleep apnea syndrome and type1/type2 diabetes mellitus: A systematic review and meta-analysis. J. Diabetes Investig..

[89] Hoang N.H., Liang Z. (2025). AI-driven sleep apnea screening with overnight blood oxygen saturation: Current practices and future directions. Front. Digit. Health.

[90] Kuo N.Y., Tsai H.J., Tsai S.J., Yang A.C. (2024). Efficient Screening in Obstructive Sleep Apnea Using Machine Learning Models: Development and Validation Study. J. Med. Internet Res..

